# Dynamic urinary proteomic analysis in a Walker 256 intracerebral tumor model

**DOI:** 10.1002/cam4.2240

**Published:** 2019-05-15

**Authors:** Linpei Zhang, Yuqiu Li, Wenshu Meng, Yanying Ni, Youhe Gao

**Affiliations:** ^1^ Department of Biochemistry and Molecular Biology Beijing Normal University, Gene Engineering Drug and Biotechnology Beijing Key Laboratory Beijing China; ^2^ Biobank The First Affiliated Hospital of Xi'an Jiaotong University Xi'an China; ^3^ Department of Pathology Aviation General Hospital of China Medical University Beijing China

**Keywords:** biomarkers, brain tumors, proteomics, urine

## Abstract

**Background:**

Patients with primary and metastatic brain cancer have an extremely poor prognosis, mostly due to the late diagnosis of disease. Urine, which lacks homeostatic mechanisms, is an ideal biomarker source that accumulates early and highly sensitive changes to provide information about the early stage of disease.

**Methods:**

A rat model mimicking the local tumor growth process in the brain was established with intracerebral Walker 256 (W256) cell injection. Urine samples were collected on days 3, 5, and 8 after injection, and then analyzed by liquid chromatography coupled with tandem mass spectrometry.

**Results:**

In the intracerebral W256 model, no obvious clinical manifestations or abnormal magnetic resonance imaging (MRI) signals were found on days 3 or 5; at these time points, 9 proteins were changed significantly in the urine of all eight tumor rats. On day 8, when tumors were detected by MRI, 25 differential proteins were identified, including 10 that have been reported to be closely related to brain metastasis or primary tumors. The differential urinary proteome was compared with those from the subcutaneous W256 model and the intracerebral C6 model. Few differential proteins overlapped, and specific differential protein patterns were observed among the three models.

**Conclusions:**

These findings demonstrate that early changes in the urine proteome can be detected in the intracerebral W256 model. The urinary proteome can reflect the difference when tumor cells with different growth characteristics are inoculated into the brain and when identical tumor cells are inoculated into different areas, specifically, the subcutis and the brain.

## 1 INTRODUCTION

Brain tumors can be divided into two main types: primary brain tumors that start within the brain, such as astrocytoma, and brain metastases, which spread from a distant primary tumor in another organ and are the most common intracranial tumor in adults.^1^, ^2^ Brain metastases occur for approximately 20%‐40% of malignant tumors, and the main metastases arise from lung cancer, breast cancer, and melanoma.^3^, ^4^ The prognosis of patients with malignant brain tumors is extremely poor, and their overall survival is significantly shortened, which seriously affects their quality of life and life expectancy.^5^ Therefore, there is an urgent need for biomarkers that can detect early brain tumors, thereby enabling reasonable and effective treatment measures for patients.

An important biomarker source, urine, is not regulated by homeostatic mechanisms, can reflect changes in the whole body, and can sensitively reflect changes caused by lesions of various organs,^6^ even the brain, which has the blood‐brain barrier. Some studies showed that urine has the potential to contain biomarkers for brain diseases.^7^ Additionally, urinary proteomics is an exciting field that provides a tool for tumor marker discovery, and new developments have occurred in recent years.^8^, ^9^ When animal models are used to evaluate urine markers, the starting point and the entire development process of the tumor can be controlled; thus, early stage urine samples can easily be collected. Moreover, the influence of genetic background, living environment, and drugs on clinical urine samples can be circumvented.

We recently demonstrated that urinary proteins could enable the early detection of cancer in a Walker 256 (W256) tumor‐bearing model using a urinary proteomics approach.^10^ In a glioma rat model injected with C6 cells, changes in urinary proteins were found earlier than magnetic resonance imaging (MRI) changes.^11^ Therefore, we asked whether inoculating the same tumor cells into different tissues would cause different urinary changes and whether different tumor cells inoculated in the same organ would cause different urinary proteome changes.

**Figure 1:**
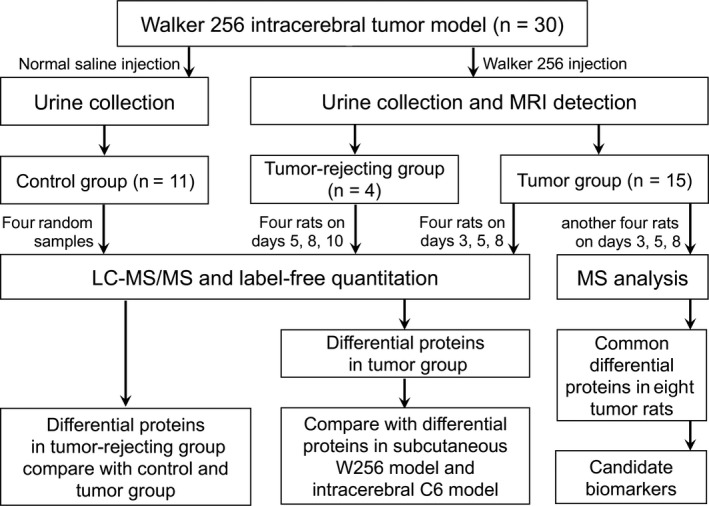
Workflow of the urinary proteome analysis in this study

In this study, a rat model was established by intracerebral injection of W256 breast carcinoma cells to mimic the local tumor growth process in the brain. Magnetic resonance imaging, the most widely used technique for diagnosing brain diseases in the clinic,^12^ was used to monitor the tumor growth. The urinary proteome in rats was analyzed after intracerebral W256 cell injection with a label‐free proteomics method by liquid chromatography coupled with tandem mass spectrometry (LC‐MS/MS). Then, the differential proteins of the intracerebral W256 model were compared with those from the subcutaneous W256 tumor‐bearing model and the intracerebral C6 glioblastoma multiforme model to investigate the ability of the urine proteome to distinguish different tumor lesions (Figure 1).

## 2 MATERIALS AND METHODS

### 2.1 Animal models

Thirty male Wistar rats (180‐200 g) were purchased from Beijing Vital River Laboratory Animal Technology Co., Ltd (Beijing, China). All animals were housed in a standard environment (12 h light/12 h dark cycle, 22 ± 1°C room temperature and 65%‐70% humidity). The experiment was approved by the Institute of Basic Medical Sciences Animal Ethics Committee, Peking Union Medical College (Animal Welfare Assurance Number: ACUC‐A02‐2014‐008) and performed according to the guidelines developed by the Institutional Animal Care and Use Committee. The W256 breast carcinoma cells were obtained from Cell Culture Center of Chinese Academy of Medical Sciences (Beijing, China).

Experimental rats (n = 19) were anesthetized with an intraperitoneal injection of a 2% sodium pentobarbital solution at 20 mg/kg and placed in a stereotaxic apparatus. A midline incision was made in the scalp, and a burr hole was drilled above the injection site (bregma: +1 mm; right 3 mm; depth 5 mm). Then, rats were injected with 5 μL of normal saline containing 2000 W256 cells using a 50‐μL microsyringe. The control rats (n = 11) were injected with 5 μL of normal saline. Urine samples were collected on days 3, 5, 8, and 10 after tumor cell injection and then stored at −80°C. During collection, rats were individually placed in metabolic cages overnight naturally for 10 hours to collect urine samples, and no water or food was provided to avoid urine contamination.

The subcutaneous W256 model and the intracerebral C6 model were used in the Wu et al^10^ and Ni et al^11^ studies, respectively. The right flanks of male Wistar rats (n = 10) were subcutaneously injected with 2 × 10^6^ viable W256 cells in 200 μL of phosphate‐buffered saline (PBS), and urine samples were collected on days 0, 4, 6, 9, and 14 after injection. The right brains of male Wistar rats (n = 20) were intracerebrally injected with 10^6^ viable C6 cells in 10 μL of PBS, and urine samples were collected on days 2, 6, 10, and 13 after injection.

### 2.2 Magnetic resonance imaging

On days 2, 5, 7, 9, and 12 after the tumor cell injection, five randomly selected rats in the experimental group were subjected to small animal MRI scans using a 7.0 Tesla PharmaScan 70/16 US (Bruker, Switzerland). Rats were anesthetized with 2.5% isoflurane. The scan sequence was T2_TurboRARE. The scanning parameters were set as follows: TR/TE = 3700/33 ms; slice thickness: 0.5 mm; field of view: 35 × 35 mm; matrix: 256 × 256; number of averages: 3; and acquisition time: 5 minutes 30 seconds.

### 2.3 Histopathology analysis

Rats were perfusion‐fixed under anesthesia with normal saline followed by 4% paraformaldehyde via the left ventricle. The brain tissues were removed and fixed in 10% formalin. Then, the tissues were embedded in paraffin and sectioned, and the pathological changes in the brain tissue after tumor cell injection were evaluated with hematoxylin and eosin staining.

### 2.4 Urine sample preparation

Urine samples were centrifuged at 12 000*g* for 30 minutes at 4°C to remove impurities and large cell debris. The supernatants were precipitated with three volumes of prechilled ethanol at −20°C for 2 hours. After centrifugation, the precipitates were dissolved in lysis buffer (8 mol/L urea, 2 mol/L thiourea, 50 mmol/L Tris, and 25 mmol/L dithiothreitol [DTT]) and then centrifuged at 12 000*g* for 30 minutes at 4°C. The protein in the resulting supernatant was quantified by the Bradford assay.

Urinary proteins were digested using the filter‐aided sample preparation method.^13^ Each 100 µg of protein was loaded onto a 10‐kDa filter device (Pall, Port Washington, NY). After sequential washing with UA buffer (8 mol/L urea, 0.1 mol/L Tris‐HCl, pH 8.5) and 25 mmol/L NH_4_HCO_3_, the proteins were reduced with 20 mmol/L DTT (Sigma) at 37°C for 1 hour and then alkylated with 50 mmol/L iodoacetamide (IAA, Sigma) in the dark for 30 minutes. Then, the samples were digested with trypsin (1:50 enzyme to protein ratio) at 37°C overnight. The resulting peptides were desalted using Oasis HLB cartridges (Waters, Milford, MA) and then dried by SpeedVac (Thermo Fisher Scientific, Waltham, MA).

### 2.5 LC‐MS/MS analysis

The digested peptides were acidified with 0.1% formic acid, and 1 μg of peptides was loaded onto a trap column (Acclaim PepMap^®^100, 75 μm × 2 cm, nanoViper C18) and separated on an analytic column (Acclaim PepMap^™ ^RSLC 100, 75 μm × 25 cm, 2 μm, nanoViper C18) using the EASY‐nLC 1200 HPLC system (Thermo Fisher Scientific). The elution gradient was 5%‐28% buffer B (0.1% formic acid in acetonitrile, flow rate = 0.3 μL/min) over 90 minutes. Peptides were analyzed using a Thermo Orbitrap Fusion Lumos Tribrid mass spectrometer (Thermo Fisher Scientific).^14^ The mass spectrometry (MS) data were acquired using the data‐dependent acquisition mode. Survey MS scans were acquired by the Orbitrap in the 350‐1550 m/z range with a resolution of 120 000. For the MS/MS scan with a resolution set to 30 000, a higher energy collision‐induced dissociation collision energy of 30 was chosen. Dynamic exclusion was employed with a 30 seconds window. Two technical replicate analyses were performed for each sample.

In the intracerebral W256 model, 4 urine samples from control rats, 12 samples on days 3, 5, and 8 from 4 randomly selected tumor rats, and 12 samples on days 5, 8, and 10 from four tumor‐rejecting rats were chosen for LC‐MS/MS analysis. In addition, the urine samples collected on days 3, 5, and 8 from another four randomly selected tumor rats and the same four control samples that were used previously, were analyzed by the same LC‐MS/MS method for biomarker validation except that the analytic columns (Acclaim PepMap^™^RSLC 100, 50 μm × 15 cm, 2 μm, nanoViper C18) and elution times (60 minutes) were different.

### 2.6 Data analysis

Raw data files from the intracerebral W256 model were searched with Mascot Daemon software (version 2.5.1, Matrix Science, London, UK) against the SwissProt_2017_02 database (taxonomy: Rattus; containing 7992 sequences) with the following parameters: trypsin digestion was selected, two sites of leaky cutting were allowed, and carbamidomethylation of cysteines was set to fixed modification. A peptide mass tolerance of 10 ppm and a fragment mass tolerance of 0.05 Da were applied. Proteins were then filtered using the decoy database method in Scaffold (version 4.7.5, Proteome Software Inc, Portland, OR). The proteins were identified with a protein false discovery rate <1%, a peptide threshold >95%, and included at least two unique peptides. The changed urinary proteins were screened with the following criteria: fold change in the increased group was ≥1.5, fold change in the decreased group ≤0.67, and *P* < 0.05 by an independent sample *t* test. The protein spectral counts of every rat in the higher group were greater than those in the lower group, and the average fold change of the spectral count in the higher group was ≥2.

Data from the Wu et al^10^ and Ni et al^11^ studies were used in this study for comparison of the different tumor models. In the intracerebral C6 model, the raw data collected by LC‐MS/MS on days 2, 6 10, and 13 from three intracerebral C6 rats and on day 2 from three control rats were reanalyzed using Scaffold for better and consistent comparison.

## 3 RESULTS

### 3.1 W256 intracerebral tumor model

**Figure 2:**
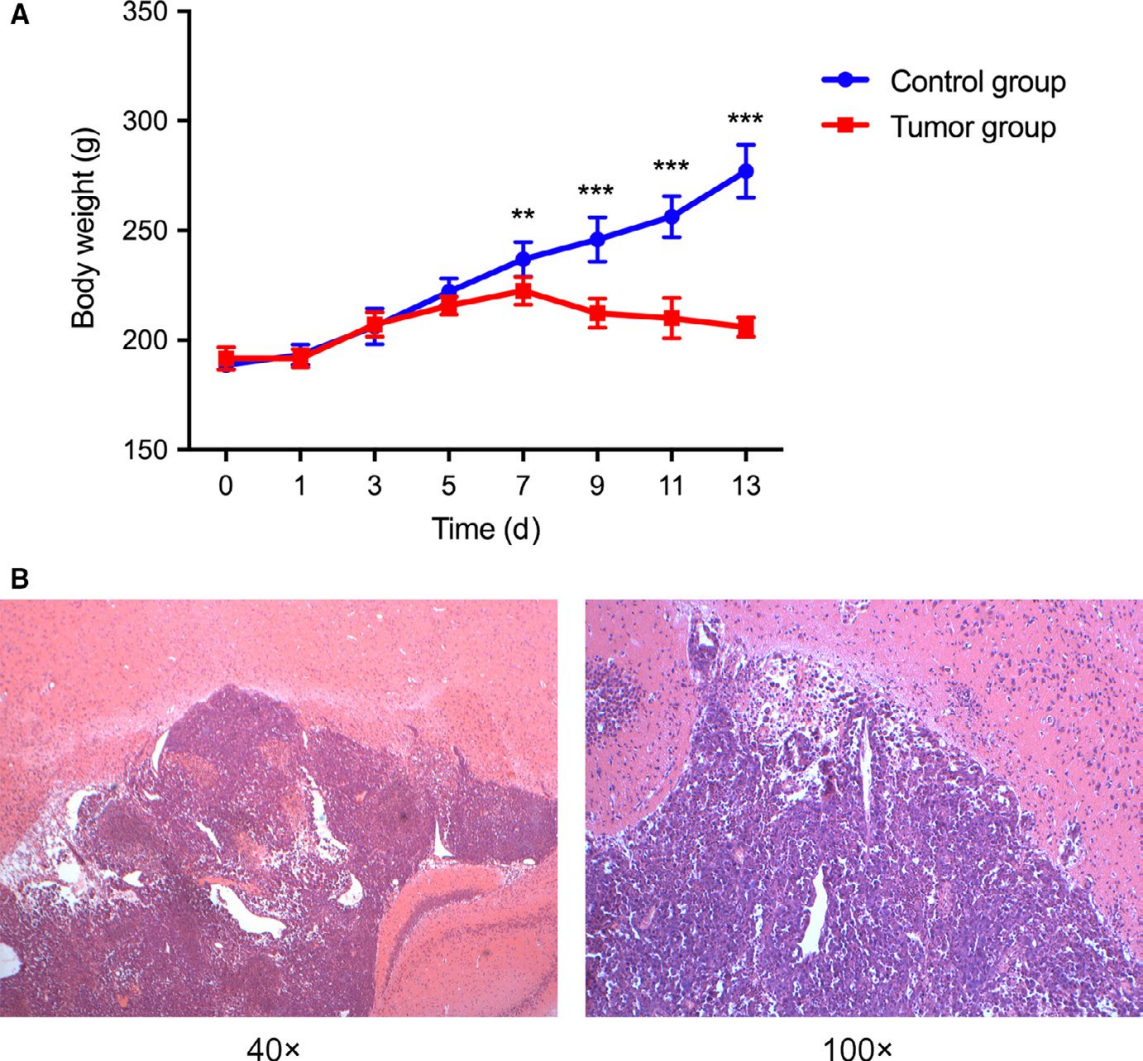
Body weights and pathological changes of the rats injected with W256 cells. A, On day 7, the rats injected with W256 cells exhibited body weights that differed significantly from those of the control group rats and began to show weight loss. *P* < 0.01 (**), *P* < 0.001 (***); B, Pathological changes in brain tissue after W256 cell injection. W256, Walker 256

On day 7, a significant difference in body weight was observed between the experimental group and the control group (Figure 2A). Furthermore, some rats showed suspected tumors on MRI. On day 9, the tumor inoculation resulted in consistent tumors in the rats, as indicated by MRI detection, except for four rats that showed no clinical signs or abnormal MRI signal on day 30 after injection. Hematoxylin and eosin staining of the brain tissue showed a large number of tumor cells, and a clear border was observable between the tumor cells and the surrounding brain parenchyma (Figure 2B).

### 3.2 MRI analysis

**Figure 3:**
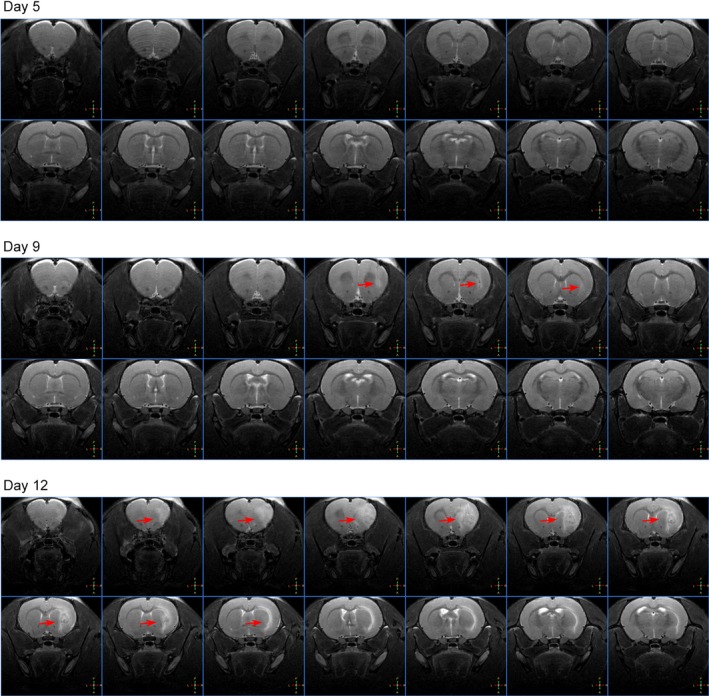
MRI changes in a rat injected with W256 cells on days 5, 9, and 12. MRI, magnetic resonance imaging

On day 5, no significant difference in tumor tissue formation existed between the right side of the brain, which was injected with tumor cells, and the left side of the brain. On day 9, a smaller tumor lesion was detected on T2‐weighted images on the right side of the brain, suggesting the presence of a tumor. As the tumor progressed, on day 12, significant tumor space‐occupying lesions and peritumoral edema were detected (Figure 3).

### 3.3 Dynamic changes in urinary proteins from intracerebral W256 rats

The urinary proteome from 4 control rat samples and 12 samples collected on days 3, 5, and 8 from 4 tumor rats were characterized by label‐free quantification to investigate the urinary protein changes with tumor progression in the brain. In total, 675 proteins were identified and are listed in Table S1. A total of 102 proteins were significantly changed in all four rats, including 21, 33, and 75 proteins on days 3, 5, and 8, respectively (Table S2).

**Figure 4:**
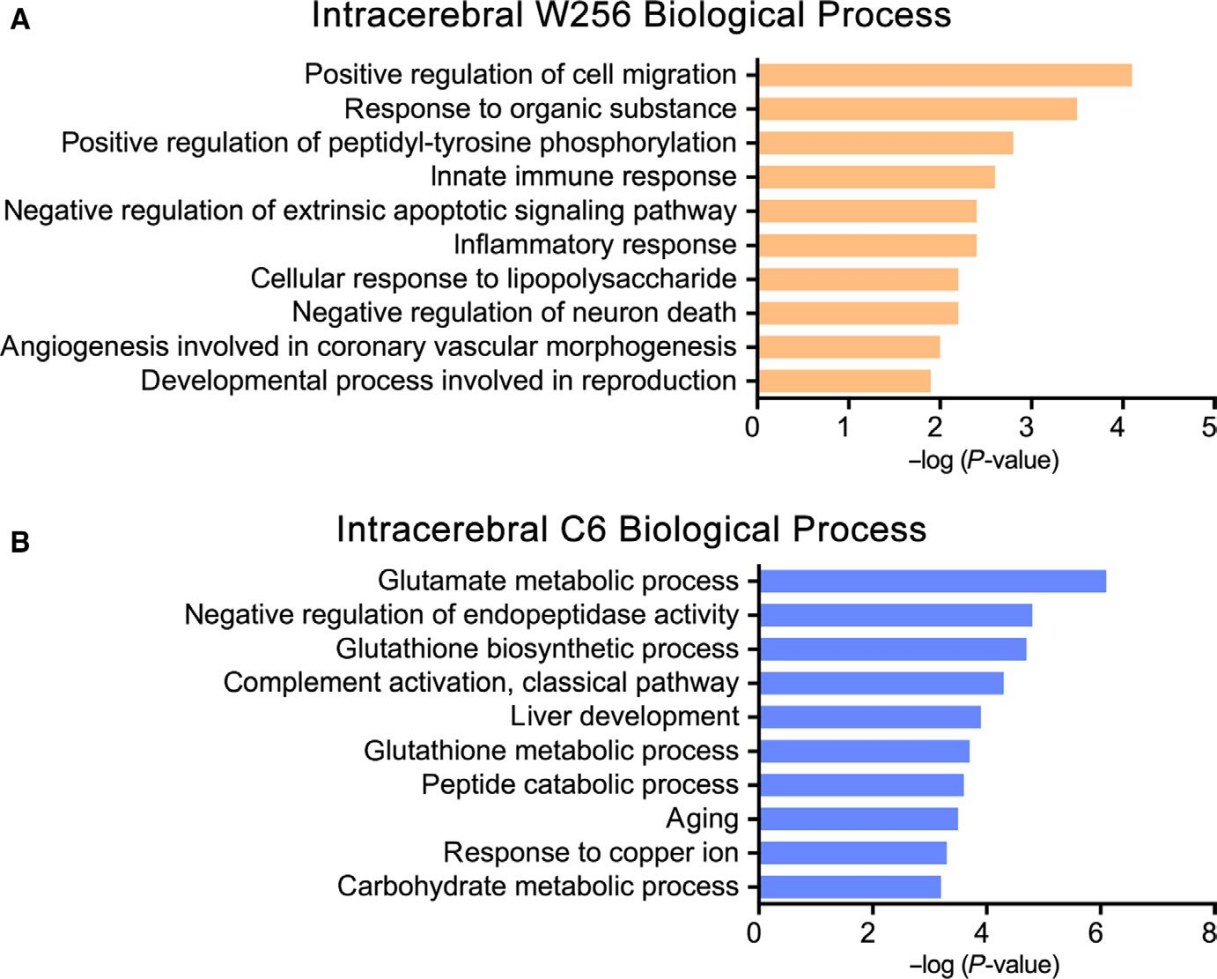
The 10 most significantly enriched biological processes. A, Changed proteins in the early stages of intracerebral W256 model; B, Changed proteins in the early stages of intracerebral C6 model. W256, Walker 256

Until the 5th day after W256 cell injection, the clinical manifestations and MRI images did not significantly differ between the experimental rats and the control rats, but 43 changed proteins were identified in urine on day 3 and day 5 (Table 1). Gene ontology (GO) analysis was performed using DAVID 6.8 (https://david.ncifcrf.gov/). Out of 43 proteins, 40 were annotated and classified into three functional categories: biological process, cellular component and molecular function. Figure 4A shows the 10 most significantly enriched biological processes. Positive regulation of cell migration, the most significant biological process is a key process for cancer cell dissemination and metastasis. The overexpression of proteins related to negative regulation of extrinsic apoptotic signaling pathway and negative regulation of neuron death may be due to the invasion and proliferation of tumor cells in the brain. In addition, extracellular exosome, extracellular space, cell surface and membrane were the most represented categories in cellular components. The most common molecular functions were receptor binding, protein homodimerization activity, growth factor binding and receptor activity.

**Table 1:** Changed urinary proteins in the early stages of intracerebral W256 model

Accession	Protein name	Human ortholog	Day 3		Day 5		Day 8	
			FC	*P*‐value	FC	*P*‐value	FC	*P*‐value
Q80WY6	Tumor necrosis factor receptor superfamily member 1B (TNFR2)	P20333	6.67	5.13E‐04	8.67	1.71E‐03	10.67	1.46E‐04
O70215	NKG2‐D type II integral membrane protein (NKG2D)	P26718	5.67	3.76E‐03	9.33	1.98E‐04	6.83	2.29E‐03
P15978	Class I histocompatibility antigen (RT1AW2)	P01891	2.35	3.76E‐03	4.50	1.24E‐03	4.50	1.50E‐03
O70513	Galectin‐3‐binding protein (LG3BP)	Q08380	3.24	3.28E‐04	3.86	1.97E‐04	1.53	1.28E‐02
P08649	Complement C4 (CO4)	P0C0L4	2.24	3.62E‐04	2.82	9.34E‐04	3.15	1.02E‐02
P07151	Beta‐2‐microglobulin (B2MG)	P61769	1.70	2.08E‐03	2.05	1.10E‐04	4.45	3.29E‐03
Q05820	Putative lysozyme C‐2 (LYZ2)	P61626	1.65	3.71E‐02	2.00	8.55E‐04	2.29	1.09E‐02
Q6DGG1	Protein ABHD14B (ABHD14B)	Q96IU4	1.60	7.63E‐03	1.53	5.75E‐03	4.57	9.69E‐03
P16391	RT1 class I histocompatibility antigen (HA12)	No	—	—	3.93	6.55E‐03	4.20	2.03E‐03
Q8JZQ0	Macrophage colony‐stimulating factor 1 (CSF1)	P09603	—	—	3.09	4.18E‐03	3.00	2.88E‐03
Q1WIM1	Cell adhesion molecule 4 (CADM4)	Q8NFZ8	—	—	2.20	1.27E‐03	2.93	1.46E‐02
P02764	Alpha‐1‐acid glycoprotein (A1AG)	P02763	—	—	1.92	1.01E‐03	4.86	8.75E‐03
P80067	Dipeptidyl peptidase 1 (DPP1)	P53634	—	—	0.60	6.48E‐03	0.57	6.28E‐04
Q9JLJ3	4‐Trimethylaminobutyraldehyde dehydrogenase (TMABADH)	P49189	—	—	0.50	1.67E‐02	0.19	2.70E‐03
Q711G3	Isoamyl acetate‐hydrolyzing esterase 1 homolog (IAH1)	Q2TAA2	—	—	0.33	1.17E‐02	0.00	1.45E‐03
Q8R5M3	Leucine‐rich repeat‐containing protein 15 (LRRC15)	Q8TF66	6.20	9.19E‐03	7.40	9.84E‐04	—	—
Q62638	Golgi apparatus protein 1 (ESL1)	Q92896	4.75	6.67E‐03	5.13	2.86E‐03	—	—
P02761	Major urinary protein (MUP)	No	0.39	3.89E‐03	0.59	1.51E‐02	—	—
P62898	Cytochrome c, somatic (CYCS)	P99999	3.33	1.63E‐03	—	—	3.83	1.85E‐03
P01946	Hemoglobin subunit alpha‐1/2 (HBA1)	P69905	8.60	4.30E‐02	—	—	—	—
P20786	Platelet‐derived growth factor receptor alpha (PDGFRA)	P16234	3.38	8.97E‐03	—	—	—	—
P16310	Growth hormone receptor (GHR)	P10912	2.71	4.42E‐03	—	—	—	—
P20762	Ig gamma‐2C chain C region (IGG2C)	No	2.25	2.45E‐03	—	—	—	—
P08753	Guanine nucleotide‐binding protein G(k) subunit alpha (GNAI3)	P08754	2.09	9.02E‐03	—	—	—	—
P08289	Alkaline phosphatase (AP‐TNAP)	P05186	1.57	9.02E‐03	—	—	—	—
P80204	TGF‐beta receptor type‐1 (TGFR‐1)	P36897	1.54	4.23E‐04	—	—	—	—
O88766	Neutrophil collagenase (MMP8)	P22894	0.54	1.05E‐02	—	—	—	—
P84855	Major urinary protein (MUP)	No	0.44	8.37E‐03	—	—	—	—
P04218	OX‐2 membrane glycoprotein (MOX2)	P41217	—	—	3.25	1.06E‐03	—	—
P49134	Integrin beta‐1 (ITGB1)	P05556	—	—	3.14	5.33E‐04	—	—
Q9Z0J6	Growth/differentiation factor 15 (GDF‐15)	Q99988	—	—	3.13	2.51E‐03	—	—
P34058	Heat shock protein HSP 90‐beta (HSP 84) (HSP84)	P08238	—	—	3.11	2.50E‐02	—	—
Q9EPF2	Cell surface glycoprotein MUC18 (MUC18)	P43121	—	—	3.08	6.82E‐03	—	—
Q9QWJ9	Neuropilin‐1 (NRP1)	O14786	—	—	2.57	3.61E‐03	—	—
P07943	Aldose reductase (AR)	P15121	—	—	2.44	1.13E‐02	—	—
P97603	Neogenin (fragment) (NEO1)	Q92859	—	—	2.10	3.63E‐04	—	—
Q80WF4	Transmembrane protein 132A (T132A)	Q24JP5	—	—	1.90	1.75E‐02	—	—
Q91XT9	Neutral ceramidase (ASAH2)	Q9NR71	—	—	1.80	1.34E‐02	—	—
Q6MG71	Choline transporter‐like protein 4 (CTL4)	Q53GD3	—	—	1.71	7.43E‐03	—	—
Q63691	Monocyte differentiation antigen CD14 (CD14)	P08571	—	—	1.70	4.12E‐03	—	—
B5DFC9	Nidogen‐2 (NID‐2)	Q14112	—	—	1.59	6.65E‐03	—	—
O35217	Multiple inositol polyphosphate phosphatase 1 (MINPP1)	Q9UNW1	—	—	1.51	1.23E‐03	—	—
O88767	Protein/nucleic acid deglycase DJ‐1 (PARK7)	Q99497	—	—	0.33	7.68E‐03	—	—

FC, fold change; — indicates failure to meet the criteria (FC ≥1.5 or ≤0.67, *P* < 0.05) compared with the control.

**Figure 5:**
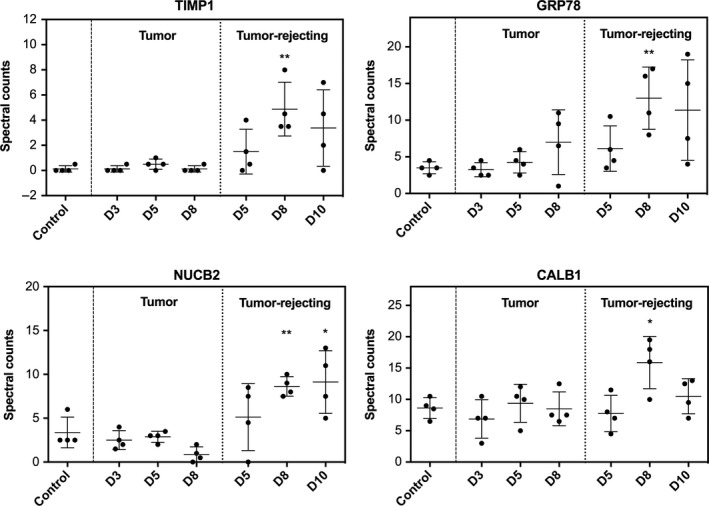
Relative quantitation of four changed urinary proteins that were significantly altered in only tumor‐rejecting rats on days 5, 8, and 10, *P* < 0.05 (*), *P* < 0.01 (**)

Interestingly, tumors did not grow in four rats after injection of highly malignant W256 cells. To investigate whether urinary protein levels can reflect some currently unclear changes in the body after W256 injection, the same quantification methods described above were used in the characterization of urinary proteome from 28 urine samples, including 4 samples from control rats, 12 samples collected on days 5, 8, and 10 from 4 tumor‐rejecting rats and 12 samples collected on days 3, 5, and 8 from four tumor rats. The identification and quantification details are shown in Table S3. Twelve, 30, and 30 proteins were altered on days 5, 8, and 10, respectively (Table S4), suggesting that the urinary proteome was changed in the tumor‐rejecting rats. We hypothesized that in addition to recovery from surgery, the urinary protein changes may be related to the eradication of a few inoculated tumor cells by the body's immune system. Some proteins, such as metalloproteinase inhibitor 1 (TIMP1), 78 kDa glucose‐regulated protein (GRP78), nucleobindin‐2 (NUCB2), and calbindin (CALB1), were found to be significantly changed in only the tumor‐rejecting rats (Figure 5). TIMP1 is an inhibitor of matrix metalloproteinases, a matrix‐degrading enzyme that facilitates tumor cell dissemination.^15^ Urinary TIMP1 was found to be a predictor of cancers, such as renal carcinoma,^16^ bladder cancer,^17^ and pancreatic malignancies.^18^ GRP78 expressed on the surface of tumor cells is associated with proliferation and metastasis.^19^ NUCB2, which is involved in breast cancer metastasis, has been reported to be differentially expressed in primary breast cancer tissues and paired metastatic lymph nodes.^20^ CALB1 was reported to prevent apoptotic death in tumor cells and is expressed in neurons.^21^ The cancer‐related proteins reflected in the urine were significantly changed in tumor‐rejecting rats but not in tumor rats, suggesting that tumor cells were indeed injected into the brain and eventually eliminated may due to the high immunity.

### 3.4 Urinary proteomic comparative analysis of three tumor models

**Figure 6:**
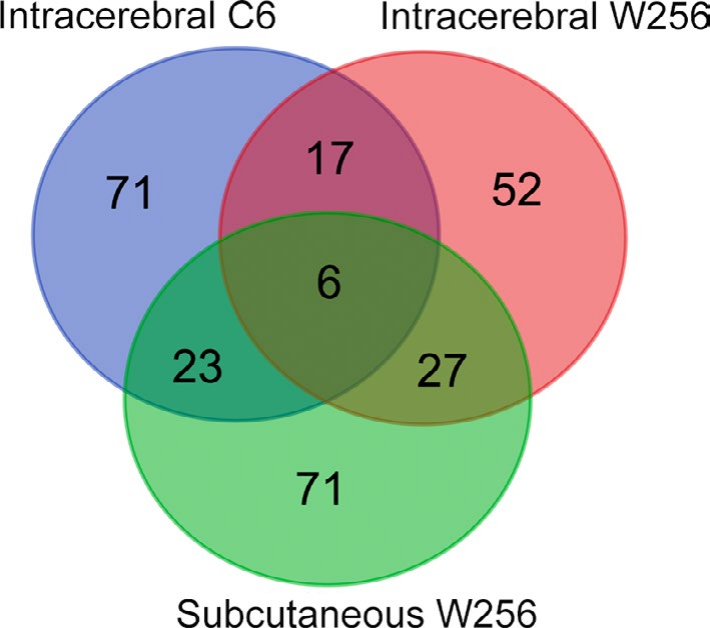
Venn diagram indicating the overlapping differential proteins in urine samples of the subcutaneous W256, intracerebral W256 and intracerebral C6 models. W256, Walker 256

In the subcutaneous W256 model, the proteome analysis results directly referred to the study published by Wu et al^10^ In the intracerebral C6 model, a total of 545 urinary proteins were identified through label‐free quantification after reanalysis (Table S5), and changed proteins on days 2, 6 10, and 13 are listed in Table S6. As shown in Figure 6, comparison of all differential proteins indicated that the proportion of overlapping proteins was small, and more than half of the differential proteins in each of the three models were unique. The details regarding the differential proteins in the three models are shown in Table S7.

Comparing the differential proteins of the intracerebral C6 and intracerebral W256 models showed only 23 overlapping proteins. Of the 23 proteins, 10 were identified in the early stages of both models, and among them, growth/differentiation factor 15, choline transporter‐like protein 4, and protein deglycase DJ‐1 were associated with nervous system function. In contrast to the intracerebral W256 model, in which a large number of differential proteins were identified at the late time point, most differential proteins could be identified at the early stages in the intracerebral C6 model. The 101 early stage differential proteins in the intracerebral C6 model were also annotated in the GO biological process analysis (Figure 4B). Glutamate metabolic process was most significantly overrepresented in the intracerebral C6 model. Glutamate is the major excitatory neurotransmitter in the central nervous system, and abnormally increased glutamate levels destroy normal tissues.^22^ These results suggest that this effect may be due to differences in the invasion abilities and patterns of W256 and C6 cells, as C6 cells diffusely infiltrated the brain parenchyma and displayed strongly invasive extension.^11^

Upon comparing the differences in urinary proteome between the intracerebral W256 model and the subcutaneous W256 model, only less than one‐third of the proteins overlapped, and the difference was greater in the late stage. In the early stages, more tumor metastasis‐associated proteins, such as beta‐2‐microglobulin (B2MG), alpha‐1‐acid glycoprotein (A1AG), galectin‐3‐binding protein (LG3BP), and macrophage colony‐stimulating factor 1 (CSF1), were identified in both the intracerebral and subcutaneous W256 models but showed no significant changes in the intracerebral C6 model. In the subcutaneous W256 model, acute‐phase response and innate immune response were significantly overrepresented at the early stage as determined by GO biological process analysis. B2MG, CSF1, and complement C4 (CO4) were enriched in the innate immune response that was also overrepresented in the intracerebral W256 model. This finding suggests that a greater number of similar urine changes will occur in the early stages when the same tumor cells are grown in different organs.

### 3.5 Validation of urinary candidate biomarkers in the intracerebral W256 model

To find more reliable urinary differential proteins associated with intracerebral tumors, the same four urine samples from control rats that were used previously and twelve urine samples collected at three time points (days 3, 5, and 8) from another four tumor rats were chosen for validation by MS analysis. As shown in Table S8, a total of 590 proteins were present in the urinary proteome, and 20, 17, and 66 changed proteins were identified on days 3, 5, and 8, respectively (Table S9). The commonly identified proteins that were significantly changed in all eight tumor rats are listed in Table 2.

**Table 2:** Differential proteins identified in eight intracerebral W256 rats

Accession	Protein name	Human ortholog	Trend^b^	Previously reported in brain tumors	
				Brain metastasis	Primary tumors
O70215	NKG2‐D type II integral membrane protein (NKG2D)^a^	P26718	↑		
O70513	Galectin‐3‐binding protein (LG3BP)^a^	Q08380	↑	Tissue^25^	
P08649	Complement C4 (CO4)^a^	P0C0L4	↑		
P07151	Beta‐2‐microglobulin (B2MG)^a^	P61769	↑	Tissue^48^	CSF^49^
Q8JZQ0	Macrophage colony‐stimulating factor 1 (CSF1)^a^	P09603	↑	Tissue^26^	
P02764	Alpha‐1‐acid glycoprotein (A1AG)^a^	P02763	↑		Serum^50^
P62898	Cytochrome c, somatic (CYCS)	P99999	↑		
Q9EPF2	Cell surface glycoprotein MUC18 (MUC18)	P43121	↑		
Q80WF4	Transmembrane protein 132A (T132A)	Q24JP5	↑		
P07632	Superoxide dismutase [Cu‐Zn] (SOD1)	P00441	↑		
Q80WY6	Tumor necrosis factor receptor superfamily member 1B (TNFR2)	P20333	↑		
P30152	Neutrophil gelatinase‐associated lipocalin (NGAL)	P80188	↑		Urine,^28^ tissue^51^
P10758	Lithostathine (REG)	P48304	↑		
Q00238	Intercellular adhesion molecule 1 (ICAM‐1)	P05362	↑	Tissue^30^	
P29534	Vascular cell adhesion protein 1 (VCAM‐1)	P19320	↑	Tissue^32^	
P50430	Arylsulfatase B (ARSB)	P15848	↓		
P00884	Fructose‐bisphosphate aldolase B (ALDOB)	P05062	↓		
P02650	Apolipoprotein E (APOE)	P02649	↓	CSF^31^	
P05544	Serine protease inhibitor A3L (SERPIN A3L)	P01011	↓		
P51635	Alcohol dehydrogenase [NADP(+)] (AKR1A1)	P14550	↓		
P04639	Apolipoprotein A‐I (APOA‐I)	P02647	↓		Tissue^52^
P50399	Rab GDP dissociation inhibitor beta (GDI‐3)	P50395	↓		
Q6P9V9	Tubulin alpha‐1B chain (TUBA1B)	P68363	↓		
P41562	Isocitrate dehydrogenase [NADP] cytoplasmic (IDH)	O75874	↓		
P19112	Fructose‐1,6‐bisphosphatase 1 (FBP1)	P09467	↓	Tissue^53^	
Q9JLJ3	4‐Trimethylaminobutyraldehyde dehydrogenase (TMABADH)	P49189	↓		
Q4KLZ6	Triokinase/FMN cyclase (TKFC)	Q3LXA3	↓		
Q9WUW9	Sulfotransferase 1C2A (ST1C2A)	O00338	↓		

a Changed proteins in the early stages continued to change differentially on day 8.

b Change trends of proteins after tumor injection compared with the control.

Among the 43 changed proteins in the early stages identified above, 9 proteins (NKG2D, LG3BP, CO4, B2MG, CSF1, A1AG, CYCS, MUC18, and T132A) that had human orthologs were also identified in the early stages of another 4 rats, and the first 6 proteins continued to change differentially on day 8. Among them, CO4, B2MG, and A1AG enable early detection of cancer according to Wu et al.^10^ LG3BP is overexpressed in the serum or tissue of patients with various types of and plays roles in cancer cell aggregation and metastasis.^23^, ^24^, ^25^ CSF1 has been reported to be expressed in brain metastases of lung cancer and breast cancer.^26^ NKG2‐D type II integral membrane protein (NKG2D) acts as an activating receptor for killer cells, providing therapeutic targets for the treatment of infectious diseases, cancer and autoimmune diseases.^27^

On day 8, the tumor rats lost weight and tumors were detectable on MRI. Among the 75 changed proteins mentioned above, 25 that had human orthologs were also identified in the other 4 rats on day 8. In addition to NKG2D, LG3BP, B2MG, CSF1, and A1AG, several proteins had been reported to be differentially expressed in the serum, urine, or brain tissue of brain metastasis or primary tumors. For instance, increased levels of neutrophil gelatinase‐associated lipocalin have been reported in the urine of patients with cancer, such as brain tumors^28^ and breast cancer.^29^ Intercellular adhesion molecule 1, a cell surface glycoprotein that is mainly expressed in nervous tissues and tumors, has been reported to be upregulated in the brain tissue of patients with lung cancer and breast cancer, and also in two breast cancer brain metastasis mouse models.^30^ Apolipoprotein E was decreased in the cerebrospinal fluid of breast cancer meningeal metastasis patients.^31^ Vascular cell adhesion protein 1 in brain metastasis‐related cerebral vessels was significantly upregulated as the tumor progressed, and this change was validated in both brain‐transferred mice and human brain tissue.^32^

## 4 DISCUSSION

Because 90% of human cancer deaths are due to metastases,^33^ the early diagnosis and prognosis of tumor metastases are very important, especially for brain metastases, the main factor limiting patients’ quality of life and life expectancy. Urine is seldom examined in biomarker research of brain metastases, in contrast with research on cerebrospinal fluid or serum. However, urine has been shown to provide clues for the early diagnosis of disease in a variety of animal models in our previous work.^34^, ^35^, ^36^, ^37^, ^38^, ^39^, ^40^, ^41^

The W256 breast carcinoma cell line has been used to construct a rat model of brain metastases that has a histopathology similar to that of patients with brain metastases.^42^, ^43^, ^44^, ^45^ Direct intracerebral injection of tumor cells can lead to tumor cell growth in the brain, which is the most well‐controlled and reproducible method for establishing animal models of brain metastases.^46^, ^47^ In this study, we identified the dynamic changes in the urine proteome in a W256 intracerebral tumor model by LC‐MS/MS analysis. Several urinary differential proteins were identified on days 3 and 5 before the appearance of obvious abnormalities on MRI. Nine proteins, including LG3BP, CSF1, and NKG2D, known to play important roles in the proliferation and metastasis of cancer, were changed significantly. When tumors were detected by MRI, 25 differential proteins were identified in the urine of all 8 tumor rats. These proteins or combinations may provide clues for the diagnosis of brain metastases, as the intracerebral W256 model mimics the local tumor growth process of brain metastases.

Notably, the urinary proteins in four tumor‐rejecting rats profiled by LC‐MS/MS showed differences compared with the tumor group, but the exact reasons underlying these differences remain unknown. Because of the limited sample size, the belief that the body's immune response cleared a small number of invaded tumor cells was only a conjecture based on the results. We suggest that the urinary proteins from the animals that were ultimately nontumorigenic after tumor cell injection merit further study. For instance, we can analyze the urinary proteins in an animal model injected with very few tumor cells.

We also compared the urinary differential proteins of the early stages among three models. The results indicated that the changes in the urine proteome are different when the same tumor cells are inoculated into different areas or different tumor cells are inoculated into the same organ. Clinically, the differential diagnosis between gliomas and single brain metastases with an unclear primary disease history is difficult because of the similar clinical and MRI manifestations, and differences exist in the diagnosis and treatment strategies between these two diseases. However, urinary proteomic comparative analysis revealed that urine proteomics may provide some new clues for definitive diagnosis.

In conclusion, urinary candidate biomarkers that have the potential to detect early tumors in the brain were identified in W256 intracerebral tumor rats. Given the small number of model animals in this preliminary study, a larger number of clinical samples are needed to validate these potential biomarkers of brain metastases in future research. Additionally, we propose that the urine proteome can provide a new direction for the diagnosis of primary tumor metastases and metastatic organs.

## CONFLICT OF INTEREST

The authors have declared that no competing interest exists.

## Data Availability

The data that support the findings of this study are openly available in figshare at http://doi.org/10.6084/m9.figshare.7831925.
